# Colonization With Multidrug-Resistant Organisms Among Healthy Adults in the Community Setting: Prevalence, Risk Factors, and Composition of Gut Microbiome

**DOI:** 10.3389/fmicb.2020.01402

**Published:** 2020-06-24

**Authors:** Yu-Shan Huang, Liang-Chuan Lai, Yu-An Chen, Kuan-Yin Lin, Yi-Hsuan Chou, Hsiu-Chi Chen, Shu-Sheng Wang, Jann-Tay Wang, Shan-Chwen Chang

**Affiliations:** ^1^Department of Internal Medicine, National Taiwan University Hospital, National Taiwan University College of Medicine, Taipei, Taiwan; ^2^Graduate Institute of Clinical Medicine, National Taiwan University College of Medicine, Taipei, Taiwan; ^3^Graduate Institute of Physiology, National Taiwan University College of Medicine, Taipei, Taiwan; ^4^Bioinformatics and Biostatistics Core, Center of Genomic and Precision Medicine, National Taiwan University, Taipei, Taiwan; ^5^Department of Medicine, National Taiwan University Hospital, New Taipei City, Taiwan; ^6^Health Management Center, National Taiwan University Hospital, Hsin-Chu, Taiwan; ^7^Department of Family Medicine, National Taiwan University Hospital, Hsin-Chu, Taiwan

**Keywords:** multidrug-resistant organisms, extended-spectrum β-lactamases, colonization, community, gut microbiota, third generation cephalosporin

## Abstract

**Background:**

The prevalence of colonization with multidrug-resistant organisms (MDROs) among healthy adults in the community is largely unknown. This study investigated the colonization rate of multidrug-resistant *Enterobacteriaceae*, methicillin-resistant *Staphylococcus aureus* (MRSA), and vancomycin-resistant enterococci (VRE) in the community in Taiwan, and compared the gut microbiota between MDRO carriers and non-carriers.

**Methods:**

This prospective cohort study was conducted from March 2017 to February 2018 at the Hsin-Chu and Jin-Shan branches of National Taiwan University Hospital. Nasal swabs and stool samples were obtained from healthy adults attending a health examination to screen for MDROs. Bacteria isolates of MDROs were tested for antibiotic susceptibility and resistant genes. Relevant data were collected using a standardized questionnaire to evaluate the risk factors for MDROs carriage, and 16S rRNA metagenomics sequencing was performed to analyze gut microbiota.

**Results:**

Among 187 participants, 4.6% (8/174) carried MRSA and 41.4% (77/186) carried third-generation cephalosporin-resistant (3GC-R) *Escherichia coli* or *Klebsiella pneumoniae*. The carriage rate of AmpC beta-lactamases and ESBL-producing strains were 16.1 and 27.4%, respectively. No carbapenem-resistant *Enterobacteriaceae* (CRE) or VRE were detected. The dominant resistant gene of *E. coli* isolates was CTX-M-type (73%), while that of *K. pneumoniae* was AmpC beta-lactamases (80%). In the multivariate analysis, the significant risk factors for carrying 3GC-R *E. coli* or *K. pneumoniae* were being an employee of technology company A [adjusted odds ratio (aOR) 4.127; 95% confidence interval (CI) 1.824–9.336; *p* = 0.001], and traveling to Southeast Asia in the past year (aOR 6.545; 95% CI 1.071–40.001; *p* = 0.042). The gut microbiota analysis showed that the phylum *Proteobacteria* and the family *Enterobacteriaceae* were significantly more abundant in 3GC-R *E. coli* and *K. pneumoniae* carriers.

**Conclusion:**

A high rate of Taiwanese adults in the community carried 3GC-R *Enterobacteriaceae*, while no CRE or VRE colonization was noted. Compared with non-carriers, an expansion of *Enterobacteriaceae* in gut microbiota was found among 3GC-R *Enterobacteriaceae* carriers.

## Introduction

The wide use of antibiotics in health care institutes and agriculture has led to the emergence of resistant pathogens (Spellberg et al., 2008; Heuer et al., 2009). As highly resistant bacteria spread worldwide, however, newer therapeutic agents are lacking. For infections caused by multidrug-resistant organisms (MDROs), effective treatment is often limited, and this is associated with additional morbidity and mortality and increased medical costs (Founou et al., 2017).

The burden of antibiotic resistance varies geographically, and is an especially great threat in Asia (Lai et al., 2014). In Taiwan, the multicenter surveillance programs for MDROs monitoring in the hospitals had revealed an increase in the prevalence of vancomycin-resistant *enterococci* (VRE) and carbapenem-resistant Gram-negative bacteria (Tseng et al., 2011). In contrast to the understanding of the epidemiology of MDROs in hospitals, the prevalence of human carriage of these resistant bacteria in the community is largely unknown. Prior studies reported that the colonization rate of methicillin-resistant *Staphylococcus aureus* (MRSA) was 3.8% and 7.8% among Taiwanese adults and children in the community, respectively (Wang et al., 2009; Chen et al., 2011). Another study surveyed *Escherichia coli* from different sources in the community setting and found that the prevalence of extended-spectrum β-lactamases (ESBL)-producers increased from 4.0 to 10.7% within 8 years (Wang et al., 2015). However, at present, data on the fecal carriage rate of VRE and carbapenem-resistant *Enterobacteriaceae* (CRE) among healthy adults in the community are still limited. It also remains unknown whether an increasing and high prevalence of MDROs in hospitals is associated with a high fecal carriage rate of these pathogens in the community.

Resistance to intestinal colonization by MDROs relies on the undisrupted commensal gut microbiota (Buffie and Pamer, 2013). It has been shown that fecal microbiota transplantation successfully eradicates colonization with MDROs in hospitalized patients (Crum-Cianflone et al., 2015). A recent study targeting VRE found that normal gut microbiota in the colon can secrete a lantibiotic to reduce VRE colonization (Kim et al., 2019). As the interaction between commensal gut microbiota and resistant pathogens were being explored, studies focusing on fecal carriage of Gram-negative bacteria and its correlation with changes in gut microbiota remained scarce.

This study aimed to investigate the prevalence of colonization with MDROs, including MRSA, third-generation cephalosporin- or carbapenem-resistant *E. coli* and *Klebsiella pneumoniae*, and VRE, among healthy adults in the community. Furthermore, differences in the gut microbiome composition between MDRO carriers and non-carriers were analyzed.

## Materials and Methods

### Study Design and Participants

This prospective cohort study was conducted at two regional hospitals (Hsin-Chu and Jin-Shan branches of National Taiwan University Hospital) in Northern Taiwan between March 2017 and February 2018. During the study period, healthy adults aged 20 years or older who attended mandatory health examination as a part of the workplace health promotion program at the two participating hospitals were invited to participate. Those who met any of the following criteria before formal enrollment were excluded: (1) being hospitalized in an acute care hospital within the past 90 days, (2) resident of a nursing home or long-term care facility, (3) received intravenous therapy, oral, or intravenous chemotherapy within the past 30 days, or (4) attended a hemodialysis clinic within the past 30 days. A total of 187 healthy adults were enrolled. After enrollment, all participants were requested to fill out a questionnaire and provide a fresh stool sample. In addition, the investigator and a well-trained study assistant took a nasal swab from each enrolled participant.

This study was approved by the Research Ethics Committee of National Taiwan University Hospital (registration No. NTUH-201711066RINA). All patients provided written informed consent before enrollment to provide a nasal swab, stool sample, and clinical data for research.

A standardized questionnaire was used to collect the participants’ demographic data, including age, sex, dietary habits, educational degree, economic status, number of household members, presence of any household member who was a health care worker, presence of any household member who was younger than 7 years old, presence of chronic diseases, smoking habits, hospitalizations within the previous year, history of caring for inpatients within the past year, outpatient clinic visits within the past year, use of antibiotics within the past year, parenteral drug use, dialysis treatment within the past year, history of animal contact, and travel history within the past year.

### Screening for MDROs

Nasal swabs were performed in both nares using the BBL^TM^ CultureSwab^TM^ (Becton, Dickinson and Company, Franklin Lakes, NJ, United States). After collection, each nasal swab was plated onto a blood agar plate. Isolates identified as *S. aureus* based on colony morphology and biochemical reactions were subcultured on CHROMID MRSA agar (BioMerieux, Marcy-l’Étoile, France) and incubated at 35°C in ambient air for 24 h to screen for MRSA. Fecal samples were collected into a clean container. A small amount of each stool sample was spread on a blood agar plate after collection, and the rest of the part was stored at −80°C as soon as possible. Cefotaxime (30 μg), ertapenem (10 μg), and imipenem (10 μg) disks were then applied to the blood agar plate and incubated at 35°C in ambient air for 24 h to screen for third-generation cephalosporin-resistant (3GC-R) *Enterobacteriaceae* or CRE. Isolates that grew within the inhibition zone of the cefotaxime and were identified as *E. coli* or *K. pneumoniae* by biochemical tests were subcultured on CHROMID ESBL agar (BioMerieux) to screen for ESBL production or not (Wickramasinghe et al., 2012; Ko et al., 2013; Blom et al., 2016). All 3GC-R *E. coli* or *K. pneumoniae*, including phenotypically selected ESBL and non-ESBL, isolates were further be tested genotypically by performing polymerase chain reaction (PCR) for the detection of AmpC β-lactamases or ESBL genes. Isolates that grew within the inhibition zone of the ertapenem and imipenem disks and were identified as *E. coli* or *K. pneumoniae* by biochemical tests were collected for further confirmation. A small amount of stool was also spread on CHROMID VRE agar (BioMerieux) to screen for VRE. Species identification of all presumed MRSA, third-generation cephalosporin- or carbapenem-resistant *E. coli* and *K. pneumoniae*, and VRE isolates were processed using the Vitek-2 bacterial identification system (BioMerieux).

### Antibiotic Susceptibility Tests

The minimal inhibitory concentration of each isolate of *S. aureus*, *E. coli*, or *K. pneumoniae* to various drugs was determined by broth microdilution using Sensititre antimicrobial susceptibility testing system with customized MRSA plate format (plate code: NHRIGP9, including cefoxitin screen test and oxacillin) and commercialized ESBL plate format (plate code: ESB1F) (Trek Diagnostic Systems, East Grinstead, United Kingdom). The interpretation of the results was performed according to Clinical and Laboratory Standards Institute 2016 criteria (CLSI, 2016). The tested drugs included oxacillin, clindamycin, erythromycin, gentamicin, ciprofloxacin, tetracycline, rifampin (rifampicin), trimethoprim-sulfamethoxazole, vancomycin, teicoplanin, daptomycin, linezolid, and tigecycline for Gram-positive bacteria, and cefoxitin, cefotaxime, ceftazidime, cefepime, gentamicin, amikacin, ciprofloxacin, imipenem, and meropenem for Gram-negative bacteria. If the Gram-negative isolates were carbapenem-resistant, the susceptibility to tigecycline and colistin was further determined. For *S. aureus* isolates, we used oxacillin MIC to detect the oxacillin resistance. For *E. coli* and *K. pneumoniae* isolates, the growth at or above the centration of cefotaxime 1 μg/mL indicated ESBL production, and an imipenem or meropenem MIC of ≥2 μg/mL indicated carbapenemase production (CLSI, 2016).

### Molecular Typing and Detection of Resistant Genes

For MRSA, multilocus sequence typing (MLST) was performed as described by Enright et al. (2000). For *Enterobacteriaceae*, multiplex PCR, which follows previously published protocols (Monstein et al., 2007; Queenan and Bush, 2007), was used to determine the presence of the genes encoding AmpC β-lactamases and ESBLs. Pulsed-field gel electrophoresis (PFGE) was used to determine the genetic relatedness among MRSA and 3GC-R *E. coli* or *K. pneumoniae*. For interpretation of the PFGE banding patterns, unweighted-pair group method using average linkages (UPGMA) dendrograms were constructed from the original data. Isolates that exhibited a similarity of 80% or greater of their banding patterns were considered to belong to the same cluster if more than three isolates were present.

### Stool DNA Extraction, 16S rRNA Gene Sequencing, and Bioinformatics Analysis

Stool genomic DNA was extracted using QIAamp DNA stool MiniKits (Qiagen, Hilden, Germany) according to the manufacturer’s instructions. The V3-V4 region of the 16S rRNA genes was amplified using universal primers linked with indices and sequencing adaptors. The amplicons were sequenced on an Illumina MiSeq platform (San Diego, CA, United States) to obtain 300-bp paired-end reads and for taxonomic assignment. The raw sequence quality was first estimated by total read counts and Q30, and then merged with Illumina Paired-End reAd mergeR (PEAR, version 0.9.8), generating effective reads. The effective reads were then denoised by QIIME (version 1.9.1), and clustered into operational taxonomic units (OTUs) with ≥ 97% sequence homology. The SILVA rRNA database (release 132) was used for taxonomy assignment of clustered OTUs. The rarefied OTU table generated by QIIME was used for calculating alpha-diversity with the Shannon diversity index and beta-diversity with weighted and unweighted UniFrac distance matrices. The UniFrac distance matrices were then used to perform principal coordinate analysis (PCoA) to compare bacterial composition differences between sample groups. The linear discriminant analysis (LDA) effect size (LEfSe) algorithm was used to identify statistically significant bacterial taxa at different taxonomic levels for MDRO carriers and non-carriers. The threshold used to consider a discriminative feature for the logarithmic LDA score was set to 3.0.

## Statistical Analysis

Categorical variables were described as proportions and compared using the Chi-squared test or Fisher’s exact test if the estimated number was less than 10. The continuous variables were described as mean ± standard deviation (SD) and compared using the Mann–Whitney U test. Univariate and multivariate logistic regression models were used to assess factors associated with the carriage of drug-resistant pathogens. All *p*-values were two-sided, and a *p*-value < 0.05 was considered statistically significant. Statistical analyses were performed using SPSS software version 25.0 (SPSS Inc., Chicago, IL, United States).

## Results

### Clinical Characteristics of the Subjects and MDROs Colonization Rates

One hundred and eighty-seven healthy adults from the community participated this study. Among whom, 174 provided nasal swabs and 186 stool samples were collected. The demographic data for all participants are presented in Table 1. The average age of the participants was 46 years, and 81 (43.3%) were male. Among all participants, 24.1% (45/187) were employees of technology company A; the other 75.9% were attendees of a health examination program who joined individually. The major products of the technology company A were the integrated circuit chip packages.

**TABLE 1:** Baseline demographics of the participants with or without fecal carriage of third-generation cephalosporin-resistant (3GC-R) *E. coli* or *K. pneumoniae*.

Baseline characteristics	All participants (*n* = 187)*	3GC-R carrier (*n* = 77)	3GC-R non-carrier (*n* = 109)	3GC-R carrier vs. non-carrier, *p*
Age (years)	39(34−63)	39(33−67)	39(34−62)	0.803
Male gender	81 (43.3)	28 (36.4)	52 (47.7)	0.124
Smoking habit**^+^**	19/173(11.0)	6/71(8.5)	12/101(11.9)	0.469
Alcohol consumption**^+^**	20/173(11.6)	8/71(11.3)	12/101(11.9)	0.902
Vegetarian**^+^**	14/171(8.2)	2/70(2.9)	12/100(12.0)	0.033
Live in dormitory**^+^**	26/175(14.9)	5/73(6.8)	21/101(20.8)	0.011
Live with family**^+^**	141/178(79.2)	61/73(83.6)	79/104(76.0)	0.221
Number of family members**^+^**	4(2−5)	4(2−6)	4(2−5)	0.684
Live with children younger than 7 years of age**^+^**	58/176(33.0)	28/73(38.4)	30/103(29.1)	0.199
**Education level**^+^****				
Below elementary school	4/177(2.3)	3/68(4.4)	1/108(0.9)	0.300
Elementary school	9/177(5.1)	2/68(2.9)	7/108(6.5)	0.485
Junior high school	16/177(9.0)	8/68(11.8)	8/108(7.4)	0.328
Senior high school	50/177(28.2)	22/68(32.4)	28/108(25.9)	0.357
College/university	72/177(40.7)	24/68(35.3)	47/108(43.5)	0.279
Postgraduate	26/177(14.7)	9/68(13.2)	17/108(15.7)	0.648
**Income (per month)**^+^****				
<20,000 NTD	42/158(26.6)	13/57(22.8)	29/100(29.0)	0.399
20,000-50,000 NTD	88/158(55.7)	34/57(59.6)	53/100(53.0)	0.420
50,000-100,000 NTD	21/158(13.3)	7/57(12.3)	14/100(14.0)	0.761
100,000-200,000 NTD	3/158(1.9)	1/57(1.8)	2/100(2.0)	>0.999
>200,000 NTD	4/158(2.5)	2/57(3.5)	2/100(2.0)	0.622
**Comorbidity**^+^****				
Hypertension	31/178(17.4)	16/73(21.9)	15/104(14.4)	0.197
Chronic hepatitis B	16/178(9.0)	6/73(8.2)	10/104(9.6)	0.750
Gastric or duodenal ulcer	10/178(5.6)	5/73(6.8)	5/104(4.8)	0.743
Diabetes mellitus	8/178(4.5)	3/73(4.1)	5/104(4.8)	0.999
Thyroid disease	7/178(3.9)	1/73(1.4)	6/104(5.8)	0.242
Urolithiasis	6/178(3.4)	1/73(1.4)	4/104(3.8)	0.650
Asthma	4/178(2.2)	2/73(2.7)	2/104(1.9)	0.999
Coronary artery disease	2/178(1.1)	0/73(0)	2/104(1.9)	0.513
Malignancy	2/178(1.1)	0/73(0)	2/104(1.9)	0.513
Travel abroad in the past year**^+^**	70/162(43.2)	26/65(40.0)	44/96(45.8)	0.464
Japan	36/162(22.2)	14/65(21.5)	22/96(22.9)	0.837
China	19/162(11.7)	5/65(7.7)	14/96(14.6)	0.184
Southeast Asia	8/162(4.9)	6/65(9.2)	2/96(2.1)	0.062
Europe	8/162(4.9)	4/65(6.2)	4/96(4.2)	0.715
Others**	7/162(4.3)	2/65(3.1)	5/96(5.2)	0.702
Animal contact in the past year**^+^**	79/167(47.3)	26/68(38.2)	53/98(54.1)	0.044
Family member as HCW**^+^**	37/169(21.9)	11/69(15.9)	26/99(26.3)	0.112
Caring for an inpatient in the past year**^+^**	21/168(12.5)	8/68(11.8)	13/99(13.1)	0.794
ER visit or hospitalization in the past year**^+^**	7/168(4.2)	5/68(7.4)	2/99(2.0)	0.122
Outpatient clinic visit in the past year**^+^**	126/165(76.3)	53/66(80.3)	73/98(74.5)	0.387
Frequency of outpatient clinic visits (per year)**^+^**	2(1−6)	3(1−10)	2(1−4)	0.187
Antibiotic use in the past year**^+^**	44/168(26.2)	21/68(30.9)	23/99(23.2)	0.270
Employee of the technology company A**^+^**	45 (24.1)	28 (36.4)	16 (14.7)	0.001

* Results are expressed as *n* (%) or median (interquartile range). 186 of 187 participants provided stool samples. ** “Others” includes: United States (*n* = 3), South Korea (*n* = 3), and Australia (*n* = 1). ^+^Data are missing for some parameters, including smoking habit (14), alcohol consumption (14), dietary habits (16), living in a dormitory (12), living with family (9), presence of household members under the age of 7 years (11), education (10), personal income (29), comorbidity (9), travel history (25), animal contact (20), family member as HCW (18), caring for an inpatient (19), ER or hospitalization (19), outpatient clinic visits (22), and antibiotic use (19). ER, emergency department; HCWs, health care workers; NTD, new Taiwan dollar.

**FIGURE 1:**
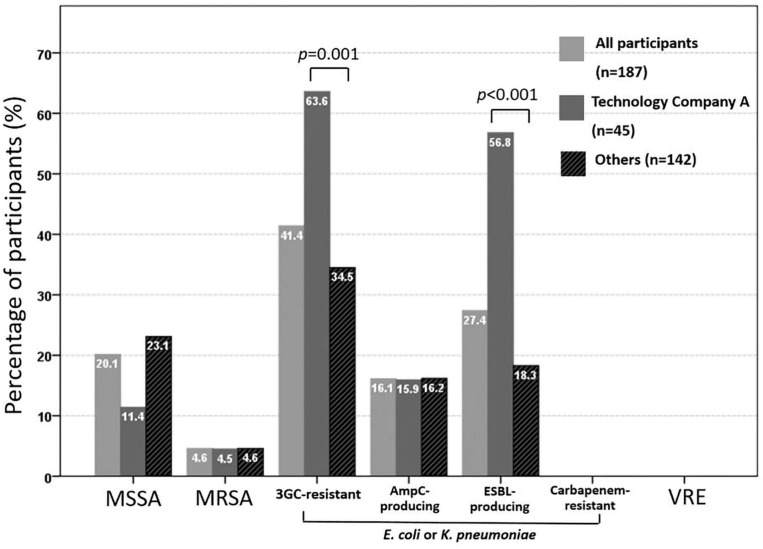
Rate of fecal colonization with third-generation cephalosporin (3GC)-resistant or carbapenem-resistant *E. coli* or *K. pneumoniae* and vancomycin-resistant enterococci (VRE) among 186 participants, and nasal colonization with methicillin-resistant *Staphylococcus aureus* (MRSA) and methicillin-sensitive *Staphylococcus aureus* (MSSA) among 174 participants.

Figure 1 shows the results of colonization with target pathogens. Overall, the prevalences of colonization with methicillin-sensitive *S. aureus*, MRSA, and 3GC-R *E. coli* or *K. pneumoniae* were 20.1% (35/174), 4.6% (8/174), and 41.4% (77/186), respectively. Using ESBL screening media, 72 participants colonized with 3GC-R *E. coli* or *K. pneumoniae* showed positivity results of ESBL screening. However, based on PCR methods, 16.1% (30/186) of the participants carried AmpC β-lactamase gene-positive isolates and 27.4% (51/186) carried ESBL-positive isolates, respectively. No fecal carriage of CRE or VRE was found. A significantly higher rate of colonization with ESBL-producing *E. coli* or *K. pneumoniae* was found among employees of technology company A than among the other participants (56.8 vs. 18.3%, respectively; *p* < 0.001), whereas the colonization rates of MRSA and AmpC β-lactamases-producing *E. coli* or *K. pneumoniae* were similar between the two groups (4.5 vs. 4.6%, *p* > 0.999 and 15.9 vs.16.2%, *p* = 0.286, respectively).

### Factors Associated With the Carriage of 3GC-R *E. coli* or *K. pneumoniae*

Comparisons of the demographics data between participants with fecal carriage of 3GC-R *E. coli* or *K. pneumoniae* (*n* = 77) and those without (*n* = 109) are shown in Table 1. In the multivariate logistic regression, the significant risk factors for carrying 3GC-R were being an employee of technology company A [adjusted odds ratio (aOR) 4.127; 95% confidence interval (CI) 1.824–9.336; *p* = 0.001] and traveling to Southeast Asia in the past year (aOR 6.545, 95% CI 1.071–40.001; *p* = 0.042) (Table 2). In the same regression model, these two factors were also significantly associated with carrying ESBL-producing *E. coli* or *K. pneumoniae*. Among the employee of technology company A, a significant higer proportion of antibiotic use in the past year was found in 3GC-R *E. coli* or *K. pneumoniae* carriers than non-carriers [10/28 (35.7%) vs. 1/16 (6.3%), *p* = 0.036] (Supplementary Table 2).

**TABLE 2:** Associated risk factors of colonization with third-generation cephalosporin-resistant *E. coli* or *K. pneumoniae*.

Variables	Univariate analysis		Multivariate analysis	
		
	Odds ratio (95% CI)	*p*	Odds ratio (95% CI)	*p*
Age, per 1 year increase	1.005 (0.987–1.023)	0.596		
Male gender	0.626 (0.345–1.138)	0.125		
Vegetarian	0.216 (0.047–0.996)	0.049		
Live in dormitory	0.280 (0.100–0.783)	0.015		
Travel to Southeast Asia in the past year	4.780 (0.934–24.470)	0.060	6.545 (1.071–40.001)	0.042
Animal contact in the past year	0.526 (0.280–0.987)	0.045		
Employee of technology company A	3.321 (1.641–6.722)	0.001	4.127 (1.824–9.336)	0.001

### Susceptibility Pattern and Genotypes of the Resistant Isolates

**FIGURE 2:**
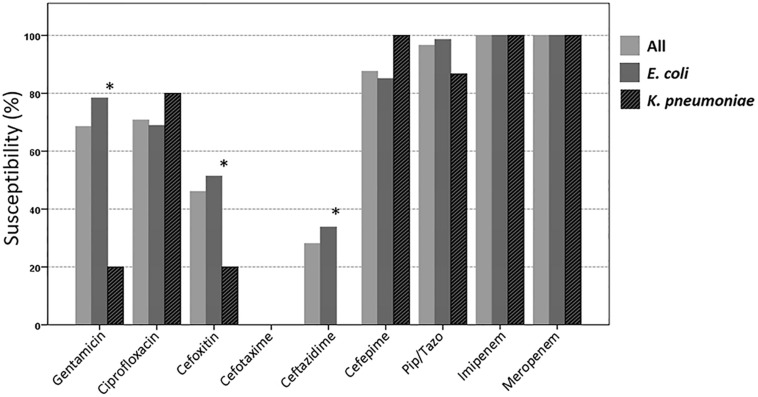
Susceptibilities of the 89 third-generation cephalosporin-resistant *E. coli* or *K. pneumoniae* isolates to different antimicrobial agents. ^∗^The susceptibility is significantly lower among *K. pneumoniae* than among *E. coli* (*p* < 0.05; Pip/Tazo, piperacillin/tazobactam).

In total, 74 3GC-R *E. coli* and 15 3GC-R *K. pneumoniae* were isolated from the stool samples of 77 participants. The susceptibilities of these isolates to piperacillin/tazobactam, cefepime, and ciprofloxacin were 96.6, 87.6, and 70.8%, respectively (Figure 2). The antibiotic susceptibility rate of *E. coli* isolates from employees of technology company A was similar to that of the other participants; however, a lower susceptibility to cefepime was found among the *E. coli* isolated from employees of technology company A (Supplementary Figure 1).

Among all 3GC-R isolates, 57 (64%) carried CTX-M-type genes, 46 (52%) non-ESBL TEM genes (all were TEM-1), 32 (36%) AmpC β-lactamase genes, 14 (16%) non-ESBL SHV-type genes, and 1 (1%) OXA-10-type gene. Two *E. coli* isolates carried both CTX-M and AmpC genes. The prevalence of CTX-M genes was significantly higher in *E. coli* than in *K. pneumoniae* (73 vs. 20%, respectively; *p* < 0.001). By contrast, AmpC genes were dominant in *K. pneumoniae*, but not in *E. coli* (80 vs. 27%, respectively; *p* < 0.001).

For the eight MRSA isolates, MLST was performed, and five sequence types were identified: ST 59 (*n* = 3), ST 508 (*n* = 2), ST 8 (*n* = 1), ST 630 (*n* = 1), and ST 2846 (*n* = 1). The susceptibilities of these MRSA isolates to different antimicrobial agents are shown in Supplementary Figure 2.

### Phylogenetic Analysis of the 3GC-R Isolates and MRSA

Phylogenetic analysis of all 3GC-R *E. coli* and *K. pneumoniae* isolates from the participants was performed using PFGE, and the results revealed great genetic diversity (Supplementary Figures 3A,B). Because of the significantly higher rate of colonization with ESBL-producers among employees of technology company A compared with the other participants, PFGE was performed with the 27 *E. coli* and three *K. pneumoniae* isolates from employees of technology company A. Only one small cluster of three *E. coli* isolates sharing ≥80% similarity in the PFGE pattern was found (Supplementary Figures 4A,B). For MRSA isolates, PFGE showed diverse pulsotypes (Supplementary Figure 5).

### Comparisons of the Gut Microbiome Between 3GC-R Carriers and Non-carriers

From participants reporting no exposure to antibiotics within the past year, 20 carrying 3GC-R *E. coli* or *K. pneumoniae* (3GC-R carriers) were randomly selected and matched to 60 non-carriers by age and sex for gut microbiota analysis. The demographic data of the 20 3GC-R carriers and 60 non-carriers are presented in Supplementary Table 1.

**FIGURE 3:**
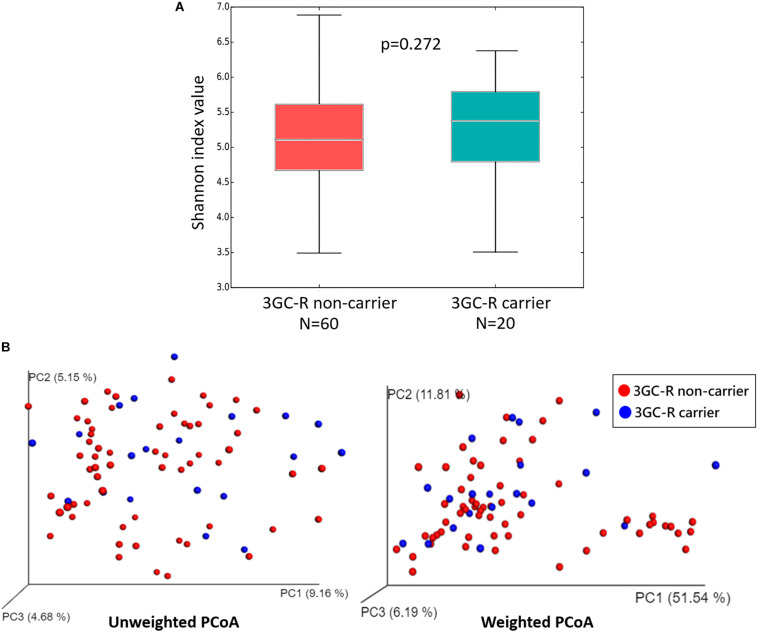
**(A)** Box plot of the alpha-diversity index measured based on the Shannon diversity index for third-generation cephalosporin-resistant (3GC-R) *E. coli* or *K. pneumoniae* carriers (blue) and non-carriers (red). Statistical testing showed no differences for the Shannon diversity index (*p* = 0.272). **(B)** PCoA plots of bacterial beta-diversity for 3GC-R carriers (blue) and non-carriers (red) based on the unweighted and weighted UniFrac distance.

The diversity within each group was measured by alpha-diversity using the Shannon diversity index, and no significant differences were found between 3GC-R carriers and non-carriers (*p* = 0.272) (Figure 3A). The diversity between the two groups (beta-diversity analysis) was compared using weighted and unweighted UniFrac analysis. The PCoA revealed that the gut microbiota of 3GC-R carriers was distinct from those of non-carriers using the unweighted UniFrac distance (*p* = 0.020), whereas the weighted UniFrac analysis was unable to differentiate carriers from non-carriers (*p* = 0.055) (Figure 3B).

**FIGURE 4:**
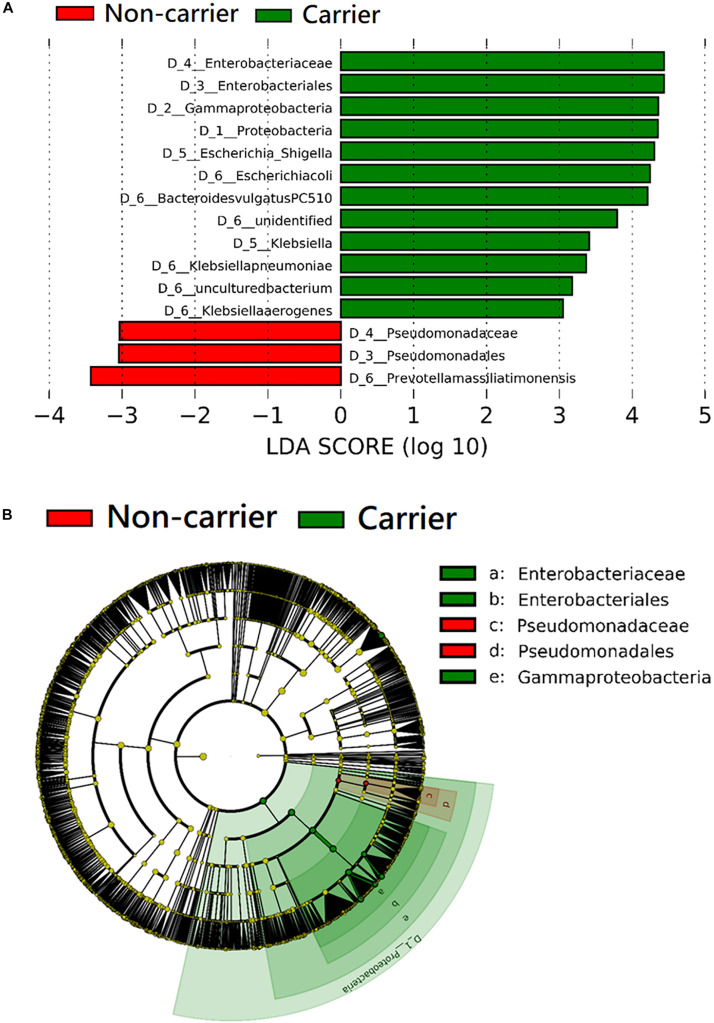
Comparison of the composition of fecal microbiota between third-generation cephalosporin-resistant *E. coli* or *K. pneumoniae* carriers (*n* = 20) and non-carriers (*n* = 60) by linear discriminant analysis (LDA) effect size (LEfSe). **(A)** Histogram of the LDA scores shows the most differentially abundant taxa in the two groups. **(B)** The cladogram shows the significantly overrepresented bacterial taxa in each group.

The taxa that differ significantly in abundance between 3GC-R carriers and non-carriers were discriminated by the LEfSe algorithm. The results of the LEfSe analysis revealed that the phylum *Proteobacteria*, as well as the family *Enterobacteriaceae* and the species *E. coli*, *K. pneumoniae*, and *Klebsiella aerogenes* were significantly more abundant in 3GC-R carriers than in non-carriers. *Bacteroides vulgatus* was also more abundant in 3GC-R carriers, while non-carriers had a higher abundance of *Prevotella massiliensis* and the family Pseudomonadaceae (Figures 4A,B).

## Discussion

In this cohort of healthy adults in the community, the rate of colonization with 3GC-R *Enterobacteriaceae* was 41.4%. No CRE or VRE colonization was noted, and the nasal carriage rate of MRSA was 4.6%, which remained stationary compared with previous reports in Taiwan. The gut microbiota analysis revealed an increased *Enterobacteriaceae* abundance in 3GC-R *E. coli* or *K. pneumoniae* carriers when compared with non-carriers.

In this study, 27.4% of healthy adults carried ESBL-producing *E. coli* or *K. pneumoniae*. The results are concordant with the high prevalence of ESBL-producing *Enterobacteriaceae* colonization reported among healthy individuals in Southeast Asia (Karanika et al., 2016; Piewngam et al., 2019). However, a study conducted in Southern Taiwan reported a low (1.9%) prevalence of ESBL-producing *E. coli* fecal carriage among healthy adults (Wu et al., 2019). This discrepancy might result from differences in local epidemiology, the proportion of participants with exposure to antibiotics, and the fact that *K. pneumoniae* was also included in our study. In Taiwan, a longitudinal study investigating 3481 *E. coli* from outpatients and patients visiting emergency rooms reported that non-susceptibility to cefotaxime reached 21.1% in 2012 (Wang et al., 2015). Another single-center study reported that 19.7% of *E. coli* isolates causing community-onset bacteremia were 3GC-R (Lin et al., 2019). Accordingly, a high prevalence of colonization with 3GC-R *Enterobacteriaceae* in the community is expected, and more extensive surveys are needed to understand the burden across Taiwan.

Several factors have been linked to increased fecal carriage of 3GC-R or ESBL-producing *Enterobacteriaceae*, including antibiotic use, international travel, hospitalization, dietary habits, and animal contact (Meyer et al., 2012; Leistner et al., 2013; Hamprecht et al., 2016; Otter et al., 2019). In our analysis, traveling to Southeast Asia in the past year and working at a specific company were associated with colonization by 3GC-R and ESBL-producing *Enterobacteriaceae*. The subgroup analysis of employees of technology company A showed that 3GC-R carriers were more likely to have antibiotics exposure in the past year. In addition, we found a higher carriage rate of ESBL-producers, but not AmpC-producers, among employees of technology company A, which indicated that the high rate of 3GC-R colonization in the community might be attributed to the spread of ESBL-producers in particular populations.

The VRE colonization rate of healthy adults is inconsistent in the literature, ranging from 0 to 21% (Balzereit-Scheuerlein and Stephan, 2001; Kolar et al., 2006; Hannaoui et al., 2016; Decker et al., 2018). Decker et al. (2018) examined 800 health care personnel and found no VRE colonization. Studies investigating CRE colonization among healthy adults in the community were scarce. One study from India reported no urine colonization of imipenem- or meropenem-resistant *Enterobacteriaceae* in 433 healthy individuals (Lohiya et al., 2015). In our study, we did not find VRE or CRE colonization among healthy adults, despite the increased prevalence of both pathogens in hospitals in Taiwan (Wang et al., 2013; Jean et al., 2018). Our results suggest that in the community, the spread of VRE or CRE occurs mainly in health care facilities such as nursing homes (Lee et al., 2017).

How commensal gut microbiota react with colonization by specific pathogens remains unclear. To date, only two studies in rural areas have investigated the composition of gut microbiota in participants with or without colonization by ESBL-producing *Enterobacteriaceae* (Gosalbes et al., 2015; Piewngam et al., 2019), and both studies included fewer cases than did our study. We showed that the gut microbiota of 3GC-R *Enterobacteriaceae* carriers was characterized by an increased relative abundance of *Proteobacteria* and *Enterobacteriaceae*; similar results had been described in CRE-carriers (Korach-Rechtman et al., 2019). The expansion of *Enterobacteriaceae* in gut microbiota is regarded as a signature of dysbiosis, which could be induced by intestinal inflammation or treatment with antibiotics (Shin et al., 2015; Litvak et al., 2017). In patients undergoing liver transplantation, the pre-transplant dysbiosis of gut microbiota has been shown to increase the likelihood of subsequent colonization by MDROs (Annavajhala et al., 2019). Our findings imply that dysbiosis gut microbiota in healthy adults is associated with colonization by drug-resistant *Enterobacteriaceae*. In humans, the domination of *Proteobacteria* or a high relative abundance of carbapenemase-producing *K. pneumoniae* in the gut microbiota has been found to increase the risk of subsequent Gram-negative rod bacteremia (Taur et al., 2012; Shimasaki et al., 2019). However, whether an increased level of *Enterobacteriaceae* in the gut microbiota would persist through the progression from asymptomatic colonization to invasive infection of drug-resistant *Enterobacteriaceae* remains unclear.

This study has several limitations. First, the number of cases was small and we included only *E. coli* and *K. pneumoniae*. The prevalence of colonization with other ESBL-producing *Enterobacteriaceae* was not evaluated. Second, the demographic data collected by the questionnaire might be imprecise and lead to recall bias. Third, the type of antibiotics taken by the participants was not documented, so we were unable to evaluate the impact of specific classes of antibiotics on the colonization of MDROs. Fourth, the number of participants from technology company A was small, which limited further statistical analysis for associated risk factors of MDRO colonization among these individuals. Lastly, the database we used in the gut microbiome analysis was unable to identify the antibiotic susceptibility profiles of each species, thus we could not know whether the increased abundance of *E. coli* in 3GC-R carriers was caused by the increase in resistant strains. It was still unknown whether subjects with MDRO fecal colonization would have an increased abundance of MDROs in their gut microbiota.

## Conclusion

In conclusion, our study shows a high prevalence of fecal carriage of 3GC-R *E. coli* or *K. pneumoniae* among healthy adults in the community, which should be considered in the empiric management of community-associated infection when involvement of *Enterobacteriaceae* is suspected. Dysbiosis of the gut microbiota presented as the expansion of *Enterobacteriaceae* was found in carriers of 3GC-R *Enterobacteriaceae*. Further studies are needed to investigate the causal relationship between changes in the gut microbiota and fecal colonization with antibiotic-resistant *Enterobacteriaceae*.

## Data Availability Statement

The datasets generated for this study can be found in the Sequence Read Archive (SRA) of NCBI with the following link: https://www.ncbi.nlm.nih.gov/sra/PRJN628533.

## Ethics Statement

The studies involving human participants were reviewed and approved by the Research Ethics Committee of National Taiwan University Hospital. The patients/participants provided their written informed consent to participate in this study.

## Author Contributions

Y-SH and J-TW: conceived and designed the analysis. Y-SH, K-YL, Y-HC, H-CC, and S-SW: acquisition of the data. Y-SH, Y-AC, L-CL, and J-TW: analysis and interpretation of the data. Y-SH: drafted the manuscript. J-TW: critical revision. S-CC: supervised the work. All authors contributed to the article and approved the submitted version.

## Conflict of Interest

The authors declare that the research was conducted in the absence of any commercial or financial relationships that could be construed as a potential conflict of interest.
