# Supplementary material for: Colonization With Multidrug-Resistant Organisms Among Healthy Adults in the Community Setting: Prevalence, Risk Factors, and Composition of Gut Microbiome
Source: Front Microbiol. 2020 Jun 24;11:1402. doi: 10.3389/fmicb.2020.01402 (PMC7328365; doi:10.3389/fmicb.2020.01402)

Supplementary Table 1 Demographics of 20 third-generation cephalosporin-resistant *E. coli* or *K. pneumoniae* carrier and 60 non-carrier with fecal microbiota analysis

| Baseline characteristics | 3GC-R carrier  (n=20) | 3GC-R non-carrier  (n=60) | *p* |
| --- | --- | --- | --- |
| Age (year) | 34.5 (32-41.5) | 36 (33-40) | 0.556 |
| Male gender | 8/20 (40.0) | 24 (40.0) | >0.999 |
| Smoking habit | 3/20 (15.0) | 10/59 (16.9) | >0.999 |
| Alcohol consumption | 2/20 (10.0) | 6/58 (10.3) | >0.999 |
| Vegetarian | 0 (0) | 0 (0) |  |
| Live in dormitory | 2/20 (10.0) | 10/59 (16.9) | 0.720 |
| Live with family | 15/20 (75.0) | 49/60 (81.7) | 0.530 |
| Number of family member | 4 (2-5) | 4 (2-6) | 0.460 |
| Live with children younger than 7 years of age | 11/20 (55.0) | 22/59 (37.3) | 0.165 |
| Education level |  |  |  |
| Below elementary school | 0/20 (0) | 1/60 (1.7) |  |
| Elementary school | 0/20 (0) | 0/60 (0) |  |
| Junior high school | 0/20 (0) | 3/60 (5.0) |  |
| Senior high school | 8/20 (40.0) | 12/60 (20.0) |  |
| College/university | 10/20 (50.0) | 32/60 (53.3) |  |
| Postgraduate | 2/20 (5.0) | 12/60 (20.0) |  |
| Income (per month) |  |  |  |
| <20,000 NTD | 3/19(15.8) | 5/59 (8.5) |  |
| 20,000-50,000 NTD | 12/19 (63.2) | 40/59 (67.8) |  |
| 50,000-100,000 NTD | 2/19 (10.5) | 13/59 (22.0) |  |
| 100,000-200,000 NTD | 0/19 (0) | 1/59 (1.7) |  |
| >200,000 NTD | 2/19 (10.5) | 0/59 (0) |  |
| Comorbidity |  |  |  |
| Hypertension | 1/20 (5.0) | 4/60 (6.7) |  |
| Chronic hepatitis B | 6/20 (30.0) | 7/60 (11.7) |  |
| Gastric or duodenal ulcer | 1/20 (5.0) | 1/60 (1.7) |  |
| Diabetes mellitus | 0/20 (0) | 0/60 (0) |  |
| Thyroid disease | 0/20 (0) | 5/60 (8.3) |  |
| Urolithiasis | 0/20 (0) | 3/60 (5.0) |  |
| Asthma | 0/20 (0) | 1/60 (1.7) |  |
| Coronary artery disease | 0/20 (0) | 0/60 (0) |  |
| Malignancy | 0/20 (0) | 0/60 (0) |  |
| Travel abroad in the past year | 7/19 (36.8) | 20/55 (36.4) | 0.970 |
| Japan | 4 | 9 |  |
| China | 2 | 4 |  |
| Southeast Asia | 1 | 1 |  |
| Europe | 0 | 3 |  |
| Others | 0 | 3 |  |
| Animal contact in the past year | 6/20 (30.0) | 37/60 (61.7) | 0.014 |
| Family member as HCW | 1/20 (5.0) | 15/60 (25.0) | 0.060 |
| Caring for an inpatient in the past year | 3/20 (15.0) | 10/60 (16.7) | >0.999 |
| ER visit or hospitalization in the past year | 1/20 (5.0) | 0 (0) | 0.250 |
| Outpatient clinic visit in the past year | 16/20 (80.0) | 44/60 (73.3) | 0.551 |
| Frequency of outpatient clinic visits (per year) | 2 (1-12) | 2 (0-4) | 0.525 |
| Employee of the technology company A | 11/20 (55.0) | 14/60 (23.3) | 0.008 |

Abbreviations: 3GC-R, third-generation cephalosporin-resistant; ER, emergency department; HCWs, health care workers; NTD, new Taiwan dollar.

Supplementary Table 2 Demographics of 44 employees of the technology company A with or without fecal carriage of third-generation cephalosporin-resistant (3GC-R) *E. coli* or *K. pneumoniae*

| Baseline characteristics | 3GC-R carrier  (n=28) | 3GC-R non-carrier  (n=16) | *p* |
| --- | --- | --- | --- |
| Age (year) | 37 (34-42) | 37 (34-40) | 0.922 |
| Male gender | 4/28 (14.3) | 2/16 (12.5) | 0.999 |
| Smoking habit | 3/28 (10.7) | 4/16 (25.0) | 0.236 |
| Alcohol consumption | 1/28 (3.6) | 3/17 (18.8) | 0.129 |
| Vegetarian | 0/28 (0) | 0/16 (0) | - |
| Live in dormitory | 1/28 (3.6) | 0/15 (0) | 0.999 |
| Live with family | 26/28 (92.9) | 14/16 | 0.614 |
| Number of family member | 4 (3-6) | 4 (3-6) | 0.202 |
| Live with children younger than 7 years of age | 13/28 (46.4) | 6/16 (37.5) | 0.565 |
| Education level |  |  |  |
| Below elementary school | 0/28 (0) | 0/16 (0) |  |
| Elementary school | 0/28 (0) | 0/16 (0) |  |
| Junior high school | 0/28 (0) | 0/16 (0) |  |
| Senior high school | 16/28 (57.1) | 9/16 (56.3) |  |
| College/university | 10/28 (35.7) | 6/16 (56.3) |  |
| Postgraduate | 2/28 (7.1) | 1/16 (6.3) |  |
| Income (per month) |  |  |  |
| <20,000 NTD | 3/28 (10.7) | 0/15 (0) |  |
| 20,000-50,000 NTD | 23/28 (82.1) | 15/15 (100) |  |
| 50,000-100,000 NTD | 0/28 (0) | 0/15 (0) |  |
| 100,000-200,000 NTD | 0/28 (0) | 0/15 (0) |  |
| >200,000 NTD | 2/28 (7.1) | 0/15 (0) |  |
| Comorbidity |  |  |  |
| Hypertension | 4/28 (14.3) | 0/16 (0) | 0.280 |
| Chronic hepatitis B | 5/28 (17.9) | 0/16 (0) | 0.141 |
| Gastric or duodenal ulcer | 2/28 (7.1) | 0/16 (0) | 0.526 |
| Diabetes mellitus | 0/28 (0) | 0/16 (0) | - |
| Thyroid disease | 0/28 (0) | 2/16 (12.5) | 0.127 |
| Urolithiasis | 0/28 (0) | 0/16 (0) | - |
| Asthma | 0/28 (0) | 0/16 (0) | - |
| Coronary artery disease | 0/28 (0) | 0/16 (0) | - |
| Malignancy | 0/28 (0) | 0/16 (0) | - |
| Travel abroad in the past year | 6/27 (22.2) | 1/13 (7.7) | 0.393 |
| Japan | 4 | 1 |  |
| China | 0 | 0 |  |
| Southeast Asia | 2 | 0 |  |
| Europe | 0 | 0 |  |
| Others | 0 | 0 |  |
| Animal contact in the past year | 8/28 (28.9) | 5/16 (31.3) | 0.999 |
| Family member as HCW | 0/28 (0) | 1/16 (6.3) | 0.364 |
| Caring for an inpatient in the past year | 4/28 (14.3) | 1/16 (6.3) | 0.638 |
| ER visit or hospitalization in the past year | 3/28 (10.7) | 0/16 (0) | 0.290 |
| Outpatient clinic visit in the past year | 20/27 (74.1) | 10/16 (62.5) | 0.502 |
| Frequency of outpatient clinic visits (per year) | 2 (0-12) | 2(0-6) | 0.579 |
| Antibiotic use in the past year | 10/28 (35.7) | 1/16 (6.3) | 0.036 |

Abbreviations: 3GC-R, third-generation cephalosporin-resistant; ER, emergency department; HCWs, health care workers; NTD, new Taiwan dollar.

Supplementary Figure 1. The susceptibility rate of the 74 *E. coli* to different antimicrobial agents between isolates from employees of the technology company A and other participants. *The susceptibility is significantly lower among employees of the technology company A. (p<0.05)


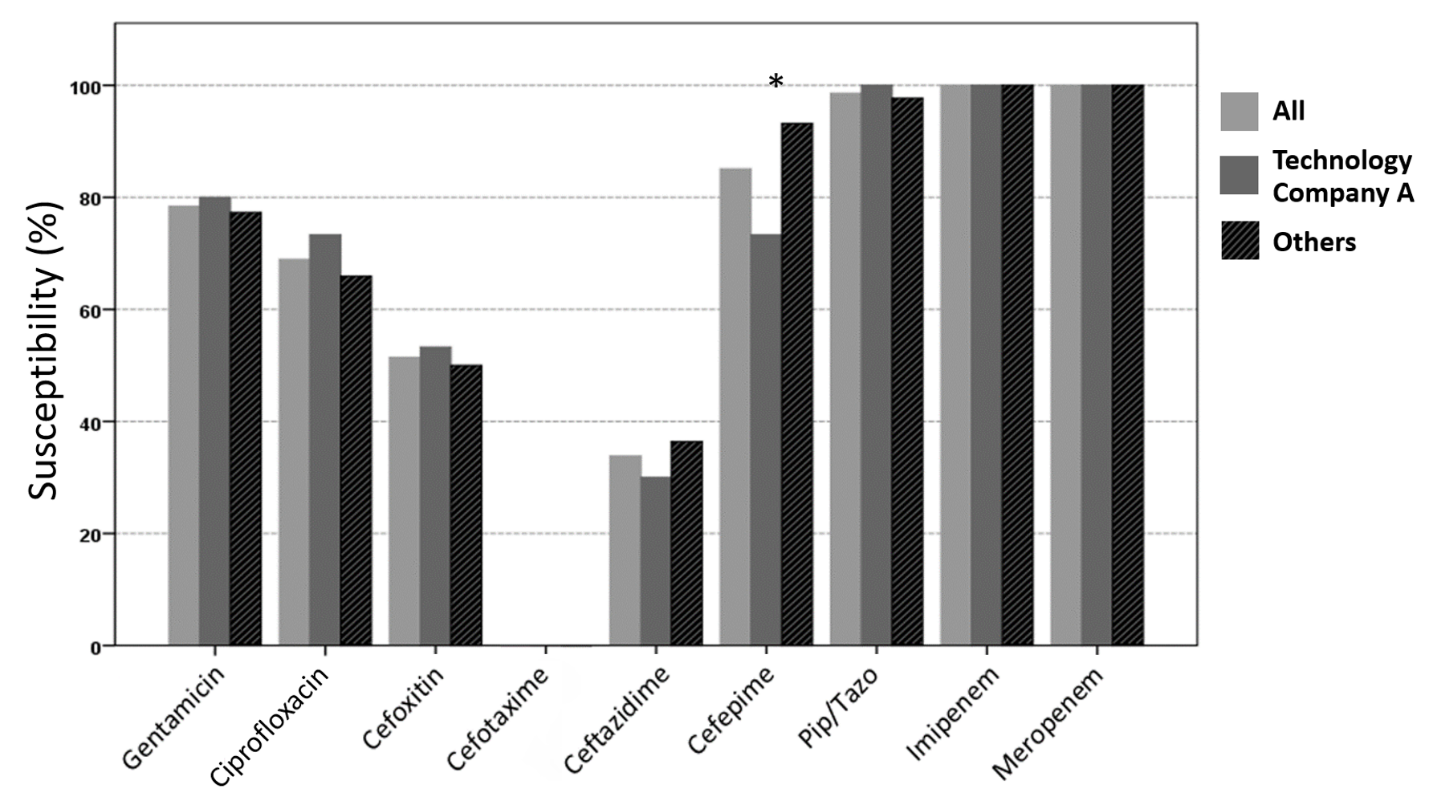


Supplementary Figure 2. The susceptibility rate of the eight MRSA isolates to different antimicrobial agents (TMP-SMX, trimethoprim-sulfamethoxazole)


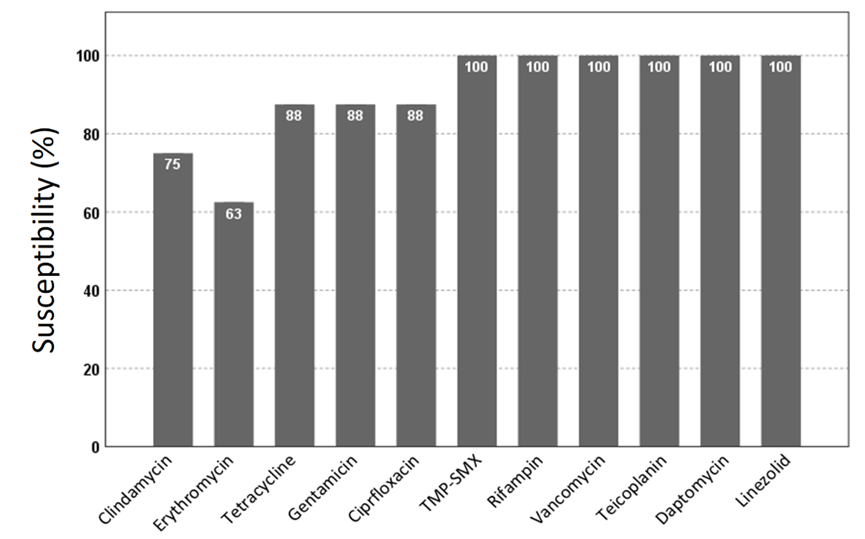


Supplementary Figure 3. Dendrogram of *Spe*I-digested genomic DNA of (A) third-generation cephalosporin-resistant *E. coli* from all participants, and (B) third-generation cephalosporin-resistant *K. pneumoniae* from all participants


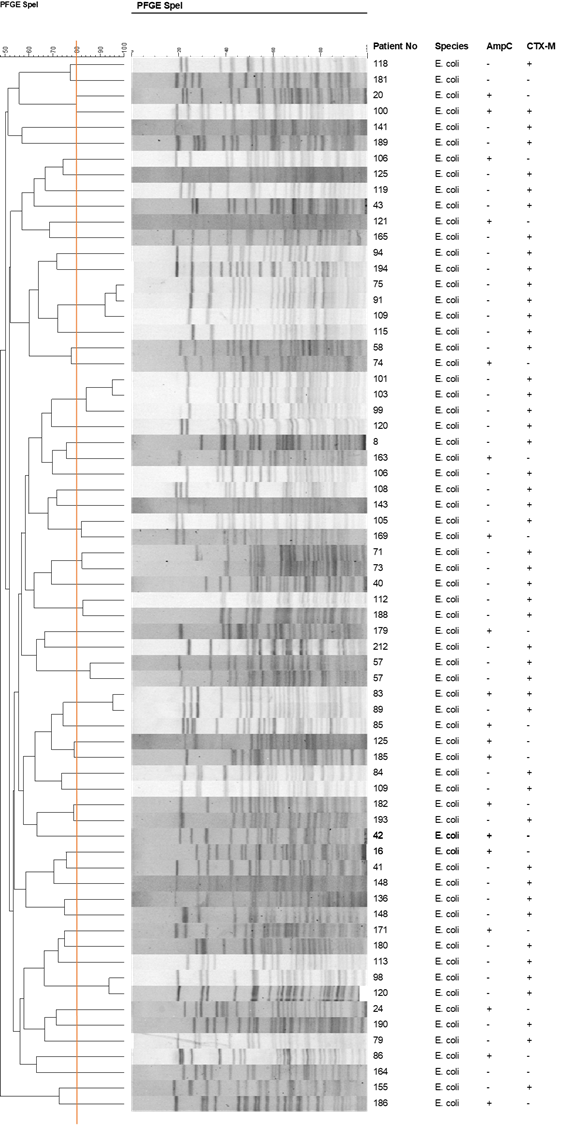
(A)

(B)


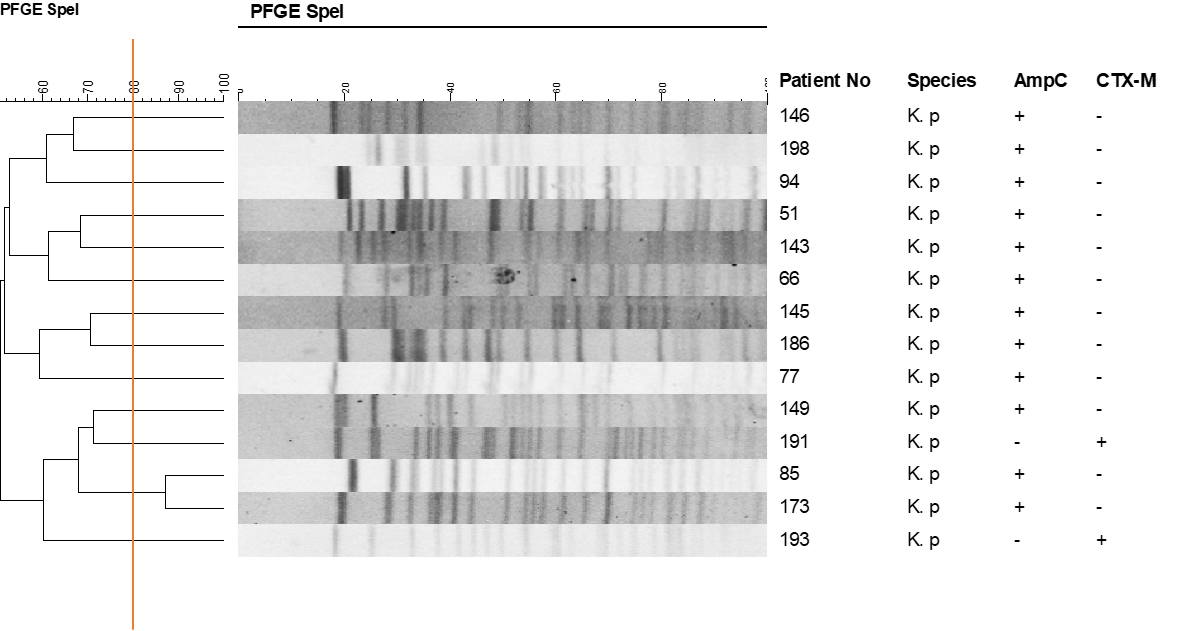


Supplementary Figure 4. Dendrogram of *Spe*I-digested genomic DNA of (A) third-generation cephalosporin-resistant *E. coli* from employees of the technology company A, and (B) third-generation cephalosporin-resistant *K. pneumoniae* from employees of the technology company A


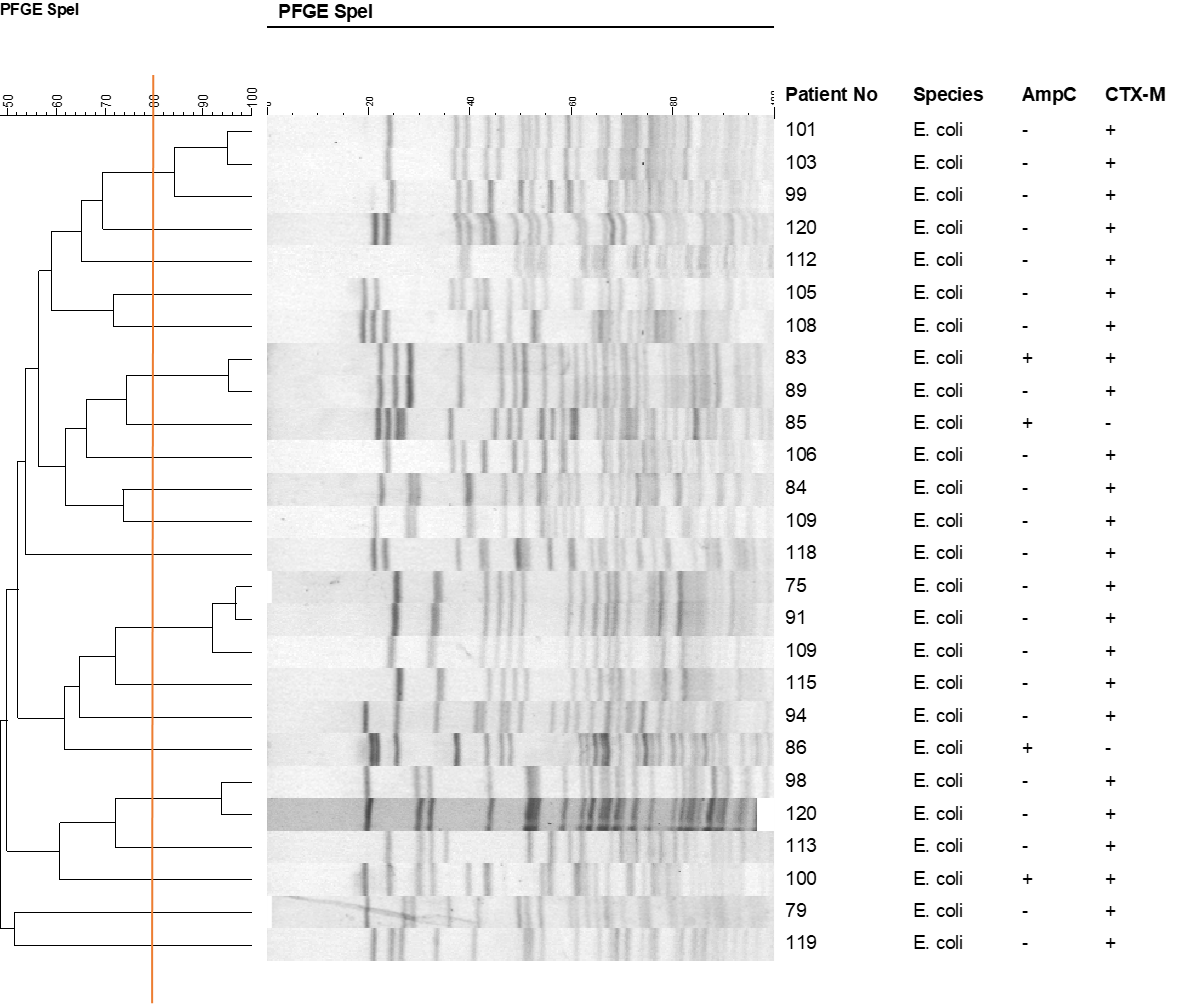
(A)

(B)


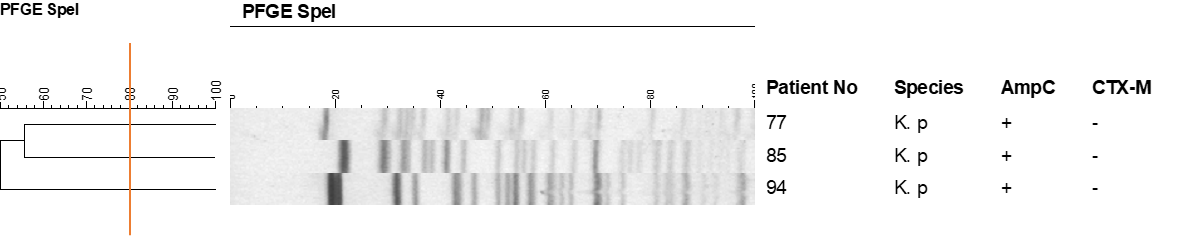


Supplementary Figure 5. Dendrogram of SmaI-digested genomic DNA of the eight Methicillin-resistant *Staphylococcus aureus* isolates


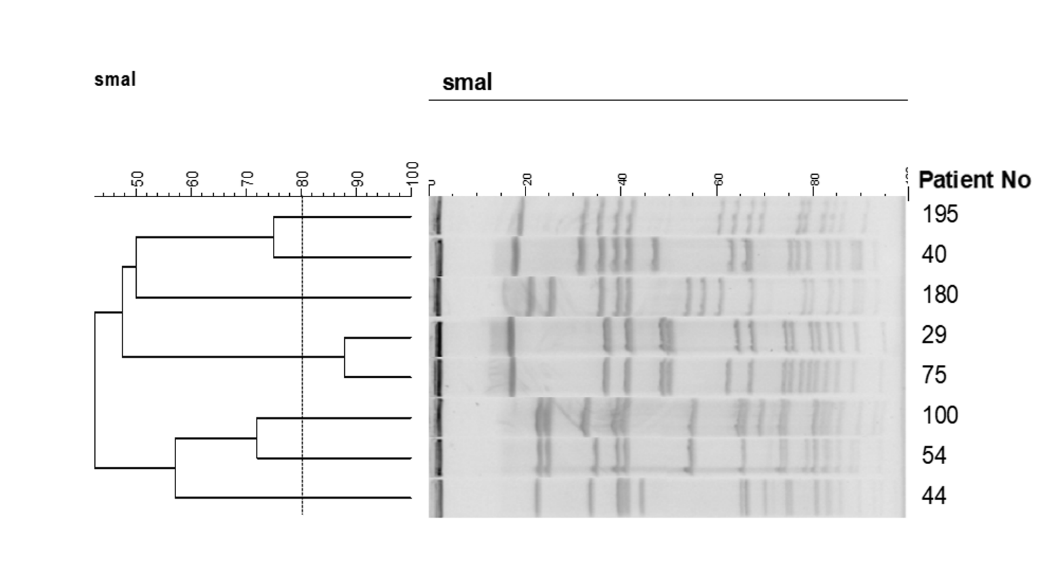

Supplement: Supplementary file 1 [file Data_Sheet_1.docx]
